# Optimal Number of Needle Punctures in EUS-FNA/B with ROSE for Solid Pancreatic Lesions

**DOI:** 10.3390/diagnostics15131692

**Published:** 2025-07-02

**Authors:** Naomi Uchiyama, Hiroshi Kawakami, Yoshinori Ozono, Hiroshi Hatada, Soichiro Ogawa, Satoshi Sekiguchi, Hiroshi Noguchi, Yuichiro Sato

**Affiliations:** 1 Division of Gastroenterology and Hepatology, Department of Internal Medicine, Faculty of Medicine, University of Miyazaki, 5200 Kihara, Kiyotake, Miyazaki 889-1692, Japan; naomi_uchiyama@med.miyazaki-u.ac.jp (N.U.); yoshinori_ohzono@med.miyazaki-u.ac.jp (Y.O.); hiroshi_hatada@med.miyazaki-u.ac.jp (H.H.); souichirou_ogawa@med.miyazaki-u.ac.jp (S.O.); 2 Center for Animal Disease Control, University of Miyazaki, Miyazaki 889-2192, Japan; sekiguchi@cc.miyazaki-u.ac.jp; 3 Department of Veterinary Science, Faculty of Agriculture, University of Miyazaki, Miyazaki 889-2192, Japan; 4 Section of Oncopathology and Morphological Pathology, Department of Pathology, Faculty of Medicine, University of Miyazaki, Miyazaki 889-1692, Japan; hiroshi_noguchi@med.miyazaki-u.ac.jp (H.N.); yuichiro_sato@med.miyazaki-u.ac.jp (Y.S.)

**Keywords:** EUS-FNA, EUS-FNB, solid pancreatic lesions, ROSE, MOSE, cumulative tissue acquisition rate, cumulative diagnostic accuracy rate

## Abstract

**Background and Objectives:** Endoscopic ultrasonography (EUS)-guided fine-needle aspiration/biopsy (FNA/B) is widely used for solid pancreatic lesions; however, the optimal number of needle punctures required to achieve high diagnostic accuracy remains unclear. This study aimed to identify the ideal number of punctures required for solid pancreatic lesions using EUS-FNA/B. **Methods:** This single-center retrospective study included 598 patients who underwent EUS-FNA/B for solid pancreatic lesions. We analyzed the cumulative tissue acquisition rates and diagnostic accuracy rates for cytology and histology, and identified the factors associated with diagnostic accuracy using univariate and multivariate analyses. Rapid on-site cytological evaluation was performed in all cases. **Results:** Cumulative tissue acquisition rates were 95.6% and 92.5% for cytology and histology, respectively. The diagnostic accuracy for cytology increased from 72.6% in the first puncture to 78.8% in the second puncture (*p* = 0.0233). In contrast, the diagnostic accuracy of histology increased from 72.0% at the first puncture to 83.2% at the third puncture (*p* = 0.0412). Statistically significant differences were noted between the first and second punctures for cytology, and between the first, second, and third punctures for histology. Univariate and multivariate analyses were conducted to identify factors associated with diagnostic accuracy. In cytology, sex was identified as a significant contributing factor, whereas no independent predictors were found in histology. **Conclusions:** These findings suggest that two-needle punctures are optimal for cytology, and three-needle punctures are optimal for the histological diagnosis of solid pancreatic lesions using EUS-FNA/B.

## 1. Introduction

Pancreatic cancer has one of the poorest prognoses, with an extremely low 5-year relative survival rate of only 11% in the USA [1]. Similarly, in Japan, the 5-year survival rate for pancreatic cancer is as low as 9.8%, likely because of challenges in its early detection [2]. Endoscopic ultrasonography (EUS) contributes to early diagnosis of pancreatic cancer. Endoscopic ultrasound-guided fine-needle aspiration/biopsy (EUS-FNA/B) is used worldwide because of its high diagnostic performance and low incidence of adverse events.

The diagnostic accuracy of EUS-FNA for pancreatic tumors has been reported in a meta-analysis to achieve a sensitivity of 84–92%, specificity of 96–98%, and diagnostic accuracy rate of 86–91%, proving its effectiveness [3,4,5]. Several meta-analyses have compared EUS-FNA with EUS-FNB for solid tumors, predominantly pancreatic tumors [6,7,8,9,10,11,12,13]. Some studies have reported that diagnostic accuracies of EUS-FNA and EUS-FNB are comparable [6,7,9,10,12], whereas others have reported on EUS-FNB in terms of diagnostic adequacy.

Cytological findings are sufficient to diagnose pancreatic adenocarcinomas. However, other diseases (such as neuroendocrine tumors, lymphoma, autoimmune pancreatitis, tuberculosis, and mass-forming chronic pancreatitis) require core tissue samples and immunohistochemical staining [14].

Based on these results, the European Society of Gastrointestinal Endoscopy (ESGE), updated in 2017 [15], recommends two–three punctures with an FNB needle to obtain a positive diagnosis if rapid on-site cytological evaluation (ROSE) cannot be performed. However, some reports have not obtained a sufficient positive diagnostic rate for solid pancreatic lesions, even after three to four punctures with an FNB needle [16,17], and the optimal number of needle punctures with EUS-FNB for the diagnosis of pancreatic lesions has not been clarified.

In recent years, tissue acquisition using third-generation biopsy needles, such as forward-facing bevel needles (Procore; COOK Medical, Bloomington, IN, USA), fork-tip needles (SharkCore; Medtronic, Dublin, Ireland), and Franseen needles (Acquire; Boston Scientific, Marlborough, MA, USA), has been widely used to treat solid pancreatic lesions. This newer-generation biopsy needle can obtain a larger number of core tissue specimens, which might lead to a better yield for a definite diagnosis [18,19]. Rodrigues-Pinto et al. reported that EUS-FNB without ROSE had a numerically higher diagnostic yield for malignancy (90%) than EUS-FNA with ROSE (77.5%), yet the difference was not statistically significant [20]. Recently, Crino et al. reported that the diagnostic accuracy of EUS-FNB using new-generation FNB needles without ROSE was comparable to that of EUS-FNB with ROSE for solid pancreatic lesions [21].

In this study, we investigated the optimal number of needle punctures for EUS-FNB of solid pancreatic lesions using ROSE.

## 2. Materials and Methods

### 2.1. Study Design

This single-center retrospective study was conducted at Miyazaki University Hospital. Data of consecutive patients who underwent EUS-FNA/B between April 2016 and July 2022 for solid pancreatic lesions were retrieved from a prospectively maintained database at Miyazaki University Hospital. The inclusion criteria were patients who underwent EUS-guided tissue acquisition using a standard 22-to-25-gauge EUS-FNB needle (Acquire needle; Boston Scientific Japan, Tokyo, Japan) or a 19-gauge EUS-FNB needle (EchoTip Ultra; Cook Medical Japan, Tokyo, Japan) for pancreatic solid lesions with available cytological and histological analyses. Patients who self-discontinued hospital visits or were undiagnosed were excluded. In cases with a clinical suspicion of benign disease, follow-up imaging was conducted at 6 months to confirm the absence of tumor growth or evidence of metastasis. Based on these findings, a diagnosis of a benign disease was established. Therefore, patients without a confirmed diagnosis of malignant disease and those who did not complete 6 months of follow-up within the study period were excluded. This study was approved by the local ethics committee at the University of Miyazaki, Japan (O-1298). Written informed consent for EUS-FNA/B was obtained from all of the patients, and informed consent for this study was obtained using the opt-out method.

### 2.2. EUS-FNA/B Procedure

EUS-FNA/B was performed using a curved linear array echoendoscope (GF-UCT260; Olympus Medical Systems, Tokyo, Japan, or EG-580-UT; Fujifilm Medical Systems, Tokyo, Japan), which was connected to a processor featuring a color Doppler function (EU-ME2; Olympus Medical Systems or SU-1, Fujifilm Medical Systems) under moderate sedation with intravenous midazolam. All FNA/B punctures were performed by experts in the EUS-FNA/B procedure with >20 years of EUS-FNA/B experience or by trainees with <5 years of EUS-FNA/B experience under the supervision of experts. During the procedure, the pancreatic mass was visualized using EUS. After careful evaluation, including assessment of the regional vasculature with color Doppler, the pancreatic head tumors were punctured via the transduodenal route, and the body/tail tumors were punctured via the transgastric route. The FNB needle with a stylet was advanced into the target lesion under EUS guidance using 10–20 mL negative syringe suction. The needle was moved to and from within the pancreatic mass approximately 20 times using the fanning technique. The obtained tissue specimens were immediately evaluated for ROSE. The number of punctures with the FNB needle was determined based on the macroscopically visible core, which was defined as white or yellow tissue with a sufficient amount of ROSE. Rapid cytological and histopathological evaluations were independently performed for each case by a minimum of two board-certified cytopathologists and pathologists, each with at least 20 years of diagnostic experience.

### 2.3. Definitions

Solid pancreatic masses confirmed by computed tomography, magnetic resonance imaging, abdominal ultrasonography, or EUS were used as evidence. For patients with pancreatic cancer, the staging classification and criteria for surgical resectability were applied according to the *Pancreatic Cancer Clinical Practice Guidelines 2022* of the Japan Pancreas Society [22].

Cytological and histological findings were classified as positive if the results indicated malignancy or suspected malignancy. Pancreatic cancer, pancreatic neuroendocrine tumors (regardless of grade), intraductal papillary mucinous carcinoma, malignant lymphoma, metastatic pancreatic tumors, and solid pseudopapillary neoplasms were considered malignant. Autoimmune pancreatitis, chronic pancreatitis, nonspecific inflammation, serous cystic neoplasms, and intraductal papillary mucinous neoplasms were considered benign. The final diagnosis of malignancy was made by a pathologist based on the surgically resected or EUS-FNA/B specimens. The final diagnosis of benign lesions was based on a negative diagnosis of malignancy using surgically resected or EUS-FNA/B specimens and no deterioration of the pancreatic lesions after at least six months of follow-up [17]. Moreover, benign disease was diagnosed at least six months after imaging evaluation to confirm the absence of tumor growth, new lesions such as enlarged surrounding lymph nodes, elevated tumor markers, or clinical symptoms.

Because this study was retrospective, the number of needle punctures varied among the patients. There were too few cases in which EUS-FNA/B was performed five or more times to allow for a meaningful analysis. Therefore, the analyses of sensitivity, specificity, positive predictive value (PPV), negative predictive value (NPV), cumulative tissue acquisition rate, and cumulative diagnostic accuracy were limited to 112 patients who underwent cytological evaluation and 108 patients who underwent histological evaluation, each with up to four EUS-FNA/B punctures.

The cumulative tissue acquisition rate was calculated by assuming that adequacy was achieved in all subsequent punctures once an adequate specimen was obtained. Similarly, cumulative diagnostic accuracy was calculated by assuming that correct diagnosis was achieved in all subsequent punctures once a correct diagnosis was made. For example, if a correct diagnosis was obtained on the first puncture, subsequent punctures (2–4) were counted as correct diagnoses regardless of whether they were truly diagnostic.

### 2.4. Statistical Analysis

The Cochran’s Q test was performed to determine the optimal number of punctures using the FNB needle. Univariate and multivariate analyses based on a logistic regression model were used to examine the factors contributing to the cytological and histological diagnostic accuracy, *p* < 0.05. To minimize the risk of overlooking potential associations due to the limited number of variables, all variables were included in both the univariate and multivariate analyses. Statistical analyses were performed using EZR (Saitama Medical Center, Jichi Medical University, Saitama, Japan), a graphical user interface for R version 1.68 (R Foundation for Statistical Computing, Vienna, Austria).

## 3. Results

### 3.1. Final Diagnosis and Patient Characteristics

**Figure 1:**
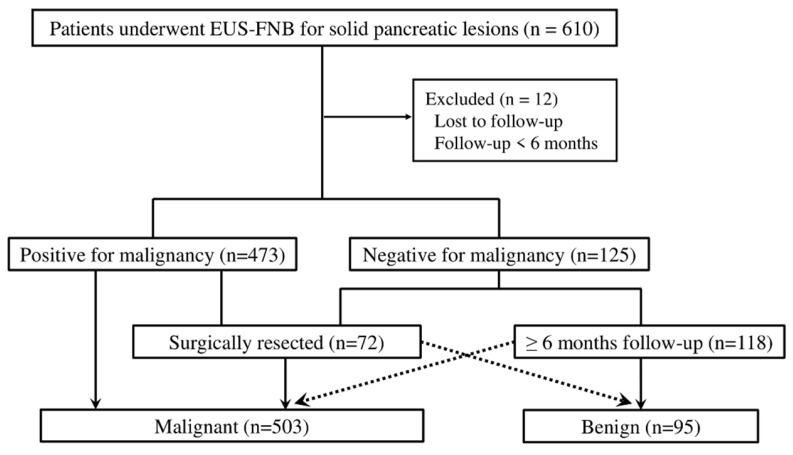
Flowchart of the study. *EUS*, endoscopic ultrasonography; *FNB*, fine-needle biopsy.

Between April 2016 and July 2022, 610 consecutive patients who underwent EUS-FNB for solid pancreatic lesions at the Miyazaki University Hospital were enrolled. Twelve of these patients were excluded from the study because they were lost to follow-up or had a follow-up period of less than 6 months. In total, 598 patients were included in this study (Figure 1). Of the 598 patients, 473 were diagnosed as positive for malignancy and 125 were diagnosed as negative using EUS-FNB specimens. Of the 598 patients, 72 underwent surgical resection, of which 71 were diagnosed as malignant and one as benign. Of the 118 patients diagnosed as negative for malignancy using EUS-FNB specimens and followed-up for more than six months, 24 were diagnosed with malignancy and 94 were diagnosed as benign. Finally, 503 patients were diagnosed with malignancy and 95 were diagnosed with benign disease (Figure 1).

Pancreatic cancer was the most common malignant tumor, accounting for 419 cases (70.1%), followed by neuroendocrine neoplasms (37 cases, 6.2%), metastatic pancreatic tumors (21 cases, 3.5%), and intraductal papillary mucinous carcinomas (12 cases, 2.0%). Autoimmune pancreatitis was the most common benign tumor, accounting for 37 cases (6.2%), followed by chronic pancreatitis in 20 cases (3.3%), and nonspecific inflammation in 14 cases (2.3%) (Table 1).

Table 2 shows the characteristics of the 598 patients enrolled in this study. The median age was 69 years (range, 15–91 years), and 344 patients (57.5%) were male. The pancreatic lesions were located in the pancreatic head in 291 patients (48.7%), tail in 145 patients (24.2%), and body in 123 patients (20.6%). The median size of the pancreatic tumor was 25.5 mm (range, 6.8–107.8). The median number of needle punctures was two (range, 1–8).

### 3.2. Optimal Number of Needle Punctures with EUS-FNB

**Figure 2:**
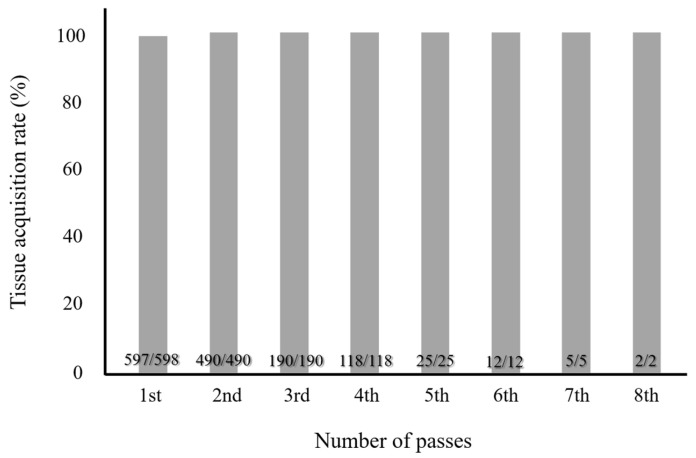
The cumulative tissue acquisition rates of EUS-FNB for solid pancreatic lesions.

The cumulative tissue acquisition rate was 99.8% for the first puncture and 100% for the second puncture in EUS-FNB of solid pancreatic lesions (Figure 2). The overall diagnostic accuracy rates for cytology, histology, and EUS-FNA/B were 93.4%, 91.4%, and 96.3%, respectively (Table 3).

**Figure 3:**
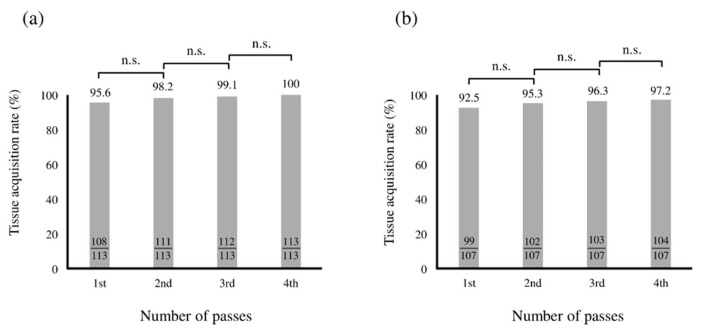
The cumulative tissue acquisition rates of EUS-FNA/B for solid pancreatic lesions in cytology (**a**) and histology (**b**). *n.s.* not significant. The cumulative tissue acquisition rates were limited to 112 patients who underwent cytological evaluation and 108 patients who underwent histological evaluation, each with up to four EUS-FNA/B punctures.

The cumulative tissue acquisition rate in cytology was 95.5% at the first puncture, and no significant difference was observed between the second and subsequent punctures (Figure 3a). Similarly, the cumulative tissue acquisition rate in histology was 92.6% in the first puncture, and no significant difference was observed between the second and subsequent punctures (Figure 3b).

**Figure 4:**
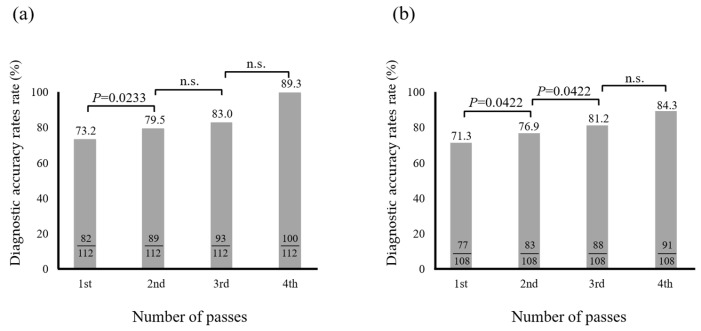
The cumulative diagnostic accuracy rates of EUS-FNB for solid pancreatic lesions in cytology (**a**) and histology (**b**). *n.s.* not significant. The cumulative diagnostic accuracy rates were limited to 112 patients who underwent cytological evaluation and 108 patients who underwent histological evaluation, each with up to four EUS-FNA/B punctures.

Next, we examined cumulative diagnostic accuracy rates. The cumulative diagnostic accuracy rates for cytology were 73.2% and 79.5% for the first and second punctures, respectively. A significant difference was observed between the first and second punctures; however, no significant difference was observed in subsequent punctures (Figure 4a). In contrast, the cumulative diagnostic accuracy rates for histology were 71.3%, 76.9%, and 81.2% for the first, second, and third punctures, respectively. There was a significant difference between the first and second punctures, and between the second and third punctures; however, no significant difference was observed between the third and fourth punctures (Figure 4b).

**Figure 5:**
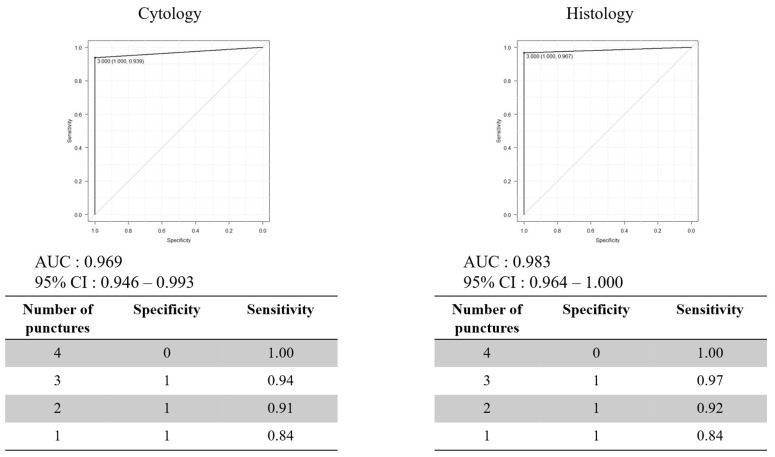
ROC curve for determining the optimal number of punctures. Three punctures as the cutoff value for both cytology and histology. AUC: area under the curve, CI: confidence interval.

The diagnostic performance of cytology and histology after each puncture is shown in Table 4. The specificity and positive predictive value of the first puncture for both cytology and histology were both 100%. The sensitivity and negative predictive value increased with the number of punctures for both cytology and histology. Receiver operating characteristic (ROC) curve analysis was performed to determine the number of needle punctures required for an accurate diagnosis. Three punctures were identified as the cut-off values for both cytology and histology (Figure 5).

The diagnostic performances of cytology and histology were limited to 112 patients who underwent cytological evaluation and 108 patients who underwent histological evaluation, each with up to four EUS-FNA/B punctures. 

### 3.3. Factors Associated with Diagnostic Accuracy

Analyses were conducted to identify factors associated with diagnostic accuracy. The univariate (*p* = 0.028) and multivariate (*p* = 0.023) analyses revealed that the diagnostic accuracy of EUS-FNA/B for solid pancreatic lesions was significantly associated with sex (Table 5a). In contrast, no independent factor was significantly associated with the diagnostic accuracy of histology (Table 5b).

## 4. Discussion

Regarding the number of punctures in EUS-FNA/B, the 2017 ESGE guidelines recommend 3–4 punctures for EUS-FNA and 2–3 punctures for EUS-FNB when on-site cytology cannot be performed. However, on-site cytology (ROSE or macroscopic on-site evaluation (MOSE)) reduces the number of punctures, while maintaining diagnostic accuracy [18,19]. Reducing the number of punctures as much as possible is beneficial for decreasing the frequency of complications, including needle-tract seeding [20]. However, no study has calculated the diagnostic or cumulative diagnostic accuracy of each puncture number to determine the optimal number of punctures. This study is innovative in its examination of the diagnostic accuracy of each puncture type in EUS-FNA/B combined with ROSE.

The overall diagnostic accuracy, regardless of the number of punctures, was 93.4% for the cytological analysis and 91.4% for the histological analysis. In a previously reported RCT [21] under similar conditions, the diagnostic accuracies were 88.6% and 92.7% for cytology and histology, respectively. Another RCT [22] involving three punctures with EUS-FNB reported a diagnostic accuracy of 83.3% for both cytology and histology. The overall diagnostic accuracy of our study was comparable to these results. In terms of diagnostic accuracy for each puncture, this study examined cases with four punctures for cytology and histology, and concluded that two punctures for cytology and three punctures for histology were optimal. The reason for obtaining diagnostic accuracy with fewer punctures in EUS-FNA than was previously reported was the use of ROSE in all cases. Performing ROSE in all medical facilities is difficult [21,23], and the effectiveness of MOSE has been reported in recent years. According to a previous randomized controlled trial, the diagnostic accuracy of EUS-guided tissue acquisition (EUS-TA) with MOSE for solid tumors was comparable to that of conventional EUS-TA without MOSE (92.6% vs. 89.3%, *p* = 0.37), although the number of needle passes was significantly lower in the MOSE group (median: 2 vs. 3, *p* < 0.001) [24]. In the present study, the overall diagnostic accuracy was 96.3%, which exceeded that of previous reports and further supports the utility of ROSE. Although a direct comparison regarding the number of punctures is limited—since previous studies included only tumors ≥20 mm—the use of MOSE may reduce the number of passes and procedure time. Therefore, MOSE may provide a valuable alternative for patients who are unsuitable for prolonged procedures. Wong et al. also reported that a diagnostic accuracy exceeding 90% was achieved for masses < 4 cm, possibly owing to the combined use of MOSE and third-generation biopsy needles designed to harvest larger core specimens [25]. Additionally, Kaneko et al. reported that in patients with pancreatic masses undergoing EUS-TA, the optimal cutoff for tissue size needed to achieve a definitive diagnosis was 10 mm (odds ratio: 5.1, *p* = 0.02). Notably, in their study, the number of needle passes was not statistically associated with diagnostic accuracy [26]. To obtain an adequate amount of tissue, Kudo et al. compared high-negative-pressure suction with conventional suction during EUS-guided tissue acquisition using a 25-gauge standard needle. They reported that the histological tissue acquisition rate was significantly higher with the high-negative-pressure technique (90%) than with the conventional method (72.2%) [27]. Furthermore, Nakai et al. reported that, compared with the conventional method, the slow-pull technique tended to show better diagnostic accuracy (odds ratio [OR], 1.48; 95% confidence interval [CI], 0.97–2.27; *p* = 0.07) and sensitivity (OR, 1.67; 95% CI, 0.95–2.93; *p* = 0.08), and was associated with a significantly lower rate of blood contamination (OR, 0.48; 95% CI, 0.33–0.69; *p* < 0.01) [28]. However, MOSE does not allow the evaluation of cellular atypia nor malignancy grade. Consequently, immediate feedback on specimen adequacy is not available, and inadequate samples may lead to repeated procedures. This can contribute to delays in diagnosis and increases procedural costs; therefore, the use of ROSE should be encouraged, when available.

Recent developments in artificial intelligence-based assessment methods have shown great promise. One study reported that by applying contrastive learning methods such as SimCLR to associate stereomicroscopic images with hematoxylin- and eosin-stained images, a diagnostic accuracy comparable to that of MOSE performed by experienced endosonographers could be achieved [29]. Further investigations with larger cohorts are required to validate these findings.

The lower cumulative diagnostic accuracy for the fourth puncture compared to the overall diagnostic accuracy is explained by the tendency of cases in which tissue acquisition is difficult or diagnosis requires three or more punctures. These results indicated that punctures beyond the fourth did not contribute to the diagnosis in a retrospective manner. One possible explanation for the lower cumulative diagnostic accuracy observed with histology than with cytology is the reduced amount of tissue remaining for histological evaluation due to the use of ROSE in all cases. Lesions requiring four EUS-FNA/B punctures are diagnostically challenging and often yield ambiguous findings on ROSE. Although inter-operator variability among cytopathologists may exist, repeated collection of samples for ROSE likely reduces the number of specimens available for histology. This may also account for the lower cumulative specimen adequacy rate observed for histology than cytology. Although ROC analysis indicated that three punctures were the optimal cut-off for both cytology and histology, histological evaluation, including immunohistochemistry, plays a critical role in diagnostically difficult cases. Therefore, we suggest limiting cytological sampling to two punctures in such cases, thereby preserving a sufficient amount of material for histological examination.

Chalhoub et al. reported that two punctures with Franseen or fork-tip needles or three punctures with any FNB needle were sufficient to achieve optimal diagnostic performance for EUS-FNB of solid pancreatic lesions, with no additional diagnostic benefit from further punctures [30]. However, ROSE was not performed on any of the patients in that study. Similarly, in the present study, we did not compare the novel biopsy needle with conventional needles. Further investigation of the performance of new biopsy needles under ROSE guidance is warranted.

Univariate and multivariate analyses were conducted to identify factors associated with diagnostic accuracy. In cytology, sex was identified as a significant contributing factor, whereas no independent predictors were found in histology. Although previous studies [31] have reported that a larger tumor size is associated with a higher diagnostic accuracy of EUS-FNA/B, our analysis demonstrated comparable diagnostic accuracy regardless of tumor size. Further prospective studies with larger sample sizes are warranted to more precisely identify factors that contribute to diagnostic accuracy.

The limitations of this study include its single-center retrospective design. Additionally, the lack of uniformity in puncture numbers and relatively small sample size pose challenges in evaluating the appropriate number of punctures. To establish evidence for the optimal number of punctures, future prospective studies with larger sample sizes, including ROSE implementation, are needed.

## 5. Conclusions

Regarding the positive diagnosis of solid pancreatic lesions with EUS-FNA/B, the optimal number of needle punctures was two for cytology and three for histology.

## Figures and Tables

**Table 1:** Final diagnosis.

Type	Diagnosis	n (%)
Malignant	Pancreatic cancer	419 (70.1)
	Neuroendocrine neoplasm	37 (6.2)
	Metastatic pancreatic tumor	21 (3.5)
	Intraductal papillary mucinous carcinoma	12 (2.0)
	Solid pseudopapillary neoplasm	5 (0.8)
	Lymphoma	5 (0.8)
	Others	3 (0.5)
	Mucinous cystic neoplasm	1 (0.2)
Benign	Autoimmune pancreatitis	37 (6.2)
	Chronic pancreatitis	20 (3.3)
	Nonspecific inflammation	14 (2.3)
	Others	14 (2.3)
	Serous cyst neoplasm	7 (1.2)
	Intraductal papillary mucinous neoplasm	3 (0.5)

Data are presented as number and percentage.

**Table 2:** Patient characteristics.

Characteristics	n = 598
Age, years	69 (15–91)
Male, sex	344 (57.5)
Lesion location	
Head	291 (48.7)
Body	123 (20.6)
Tail	145 (24.2)
Head/body	9 (1.5)
Body/tail	22 (3.7)
Uncinate	8 (1.3)
Tumor size, mm	25.5 (6.8–107.8)
Number of passes	2 (1–8)

Data are presented as median (range) or number (%).

**Table 3:** The overall diagnostic performance of the cytology and histology.

	n (%)
Cytology	554 (93.4)
Histology	540 (91.4)
Cytology ± Histology	576 (96.3)

**Table 4:** The diagnostic performance of the cytology and histology.

	Cytology				Histology			
	1st	2nd	3rd	4th	1st	2nd	3rd	4th
Sensitivity	73.3	80	83.8	90.5	70.3	81.2	87.1	87.1
Specificity	100	100	100	100	100	100	100	100
PPN	100	100	100	100	100	100	100	100
NPV	20	29	29.2	41.2	18.9	26.9	39	35

PPV: positive predictive value, NPV: negative predictive value.

**Table 5:** (**a**) Factors associated with cytological diagnostic accuracy. (**b**) Factors associated with histological diagnostic accuracy.

	Univariate Analysis		Multivariate Analysis	
Variables	OR (95% CI)	*p*-Value	OR (95% CI)	*p*-Value
(**a**)				
Age ≥ 70 years	1.7 (0.5–5.3)	0.394	1.6 (0.48–5.4)	0.439
Male	0.2 (0.06–0.85)	0.028	2.1 (0.05–0.81)	0.023
Malignant	1.5 (0–Inf)	0.99	6.2 (0–Inf)	0.994
Diameter ≥ 10 mm	1.6 (0–Inf)	0.99	9.3 (0–Inf)	0.996
Location (head)	0.8 (0.3–2.4)	0.67	6.7 (0.2–2.2)	0.5
(**b**)				
Age ≥ 70 years	1.0 (0.4–2.6)	1.0	1.2 (0.4–3.4)	0.731
Male	0.7 (0.3–1.9)	0.498	0.8 (0.3–2.2)	0.607
Malignant	0.5 (0.06–3.9)	0.476	1.1 (0.2–5.3)	0.952
Diameter ≥ 10 mm	1.4 (0.14–13)	0.79	1.7 (0.2–18)	0.665
Location (head)	1.3 (0.5–3.6)	0.558	1.2 (0.4–3.4)	0.759

OR, odds ratio; CI, confidence interval.

## Data Availability

Data described in this manuscript are available from the corresponding author upon request.

## References

[1] Siegel R.L., Miller K.D., Fuchs H.E., Jemal A. (2022). CA: A Cancer Cancer Stat. J. Clin..

[2] Cancer Research Foundation: Cancer Statistics 2021. https://ganjoho.jp/public/qa_links/report/statistics/pdf/cancer_statistics_2021.pdf.

[3] Banafea O., Mghanga F.P., Zhao J., Zhao R., Zhu L. (2016). Endoscopic ultrasonography with fine-needle aspiration for histological diagnosis of solid pancreatic masses: A meta-analysis of diagnostic accuracy studies. BMC Gastroenterol..

[4] Puli S.R., Bechtold M.L., Buxbaum J.L., Eloubeidi M.A. (2013). How good is endoscopic ultrasound-guided fine-needle aspiration in diagnosing the correct etiology for a solid pancreatic mass?: A meta-analysis and systematic review. Pancreas.

[5] Yang Y., Li L., Qu C., Liang S., Zeng B. (2016). Endoscopic ultrasound-guided fine needle core biopsy for the diagnosis of pancreatic malignant lesions: A systematic review and Meta-Analysis. Sci. Rep..

[6] Khan M.A., Grimm I.S., Ali B., Nollan R., Tombazzi C., Ismail M.K., Baron T.H. (2017). A meta-analysis of endoscopic ultrasound-fine-needle aspiration compared to endoscopic ultrasound-fine-needle biopsy: Diagnostic yield and the value of onsite cytopathological assessment. Endosc. Int. Open.

[7] Li Z., Liu W., Xu X., Li P. (2022). A meta-analysis comparing endoscopic ultrasound-guided fine-needle aspiration with endoscopic ultrasound-guided fine-needle biopsy. J. Clin. Gastroenterol..

[8] van Riet P.A., Erler N.S., Bruno M.J., Cahen D.L. (2021). Comparison of fine-needle aspiration and fine-needle biopsy devices for endoscopic ultrasound-guided sampling of solid lesions: A systemic review and meta-analysis. Endoscopy.

[9] Facciorusso A., Bajwa H.S., Menon K., Buccino V.R., Muscatiello N. (2020). Comparison between 22G aspiration and 22G biopsy needles for EUS-guided sampling of pancreatic lesions: A meta-analysis. Endosc. Ultrasound.

[10] Facciorusso A., Bajwa H.S., Menon K., Buccino V.R., Muscatiello N. (2019). Comparative accuracy of needle sizes and designs for EUS tissue sampling of solid pancreatic masses: A network meta-analysis. Gastrointest. Endosc..

[11] Li H., Li W., Zhou Q.Y., Fan B. (2018). Fine needle biopsy is superior to fine needle aspiration in endoscopic ultrasound guided sampling of pancreatic masses: A meta-analysis of randomized controlled trials. Medicine.

[12] Wang J., Zhao S., Chen Y., Jia R., Zhang X. (2017). Endoscopic ultrasound guided fine needle aspiration versus endoscopic ultrasound guided fine needle biopsy in sampling pancreatic masses: A meta-analysis. Medicine.

[13] Renelus B.D., Jamorabo D.S., Boston I., Briggs W.M., Poneros J.M. (2021). Endoscopic ultrasound-guided fine needle biopsy needles provide higher diagnostic yield compared to endoscopic ultrasound-guided fine needle aspiration needles when sampling solid pancreatic lesions: A meta-analysis. Clin. Endosc..

[14] Park J.K., Kang K.J., Oh C.R., Lee J.K., Lee K.T., Jang K.T., Park S.M., Lee K.H. (2016). Evaluating the minimal specimens from endoscopic ultrasound-guided fine-needle aspiration in pancreatic masses. Medicine.

[15] Polkowski M., Jenssen C., Kaye P., Carrara S., Deprez P., Gines A., Fernández-Esparrach G., Eisendrath P., Aithal G.P., Arcidiacono P. (2017). Technical aspects of endoscopic ultrasound (EUS)-guided sampling in gastroenterology: European society of gastrointestinal endoscopy (ESGE) technical guideline-march 2017. Endoscopy.

[16] Cheng B., Zhang Y., Chen Q., Sun B., Deng Z., Shan H., Dou L., Wang J., Li Y., Yang X. (2018). Analysis of fine-needle biopsy vs fine-needle aspiration in diagnosis of pancreatic and abdominal masses: A Prospective, multicenter, randomized controlled trial. Clin. Gastroenterol. Hepatol..

[17] Tian L., Tang A.L., Zhang L., Liu X.W., Li J.B., Wang F., Shen S.R., Wang X.Y. (2018). Evaluation of 22G fine-needle aspiration (FNA) versus fine-needle biopsy (FNB) for endoscopic ultrasound-guided sampling of pancreatic lesions: A prospective comparison study. Surg. Endosc..

[18] Imaoka H., Sasaki M., Hashimoto Y., Watanabe K., Miyazawa S., Shibuki T., Mitsunaga S., Ikeda M. (2021). Impact of endoscopic ultrasuound-guided tissue acquisition on decision-making in precision medicine for pancreatic cancer: Beyond diagnosis. Diagnostics..

[19] Oppong K.W., Bekkali N.L.H., Leeds J.S., Johnson S.J., Nayar M.K., Darné A., Egan M., Bassett P., Haugk B. (2020). Fork-tip needle biopsy versus fine-needle aspiration in endoscopic ultrasound-guided sampling of solid pancreatic masses; a randomized crossover study. Endoscopy.

[20] Rodrigues-Pinto E., Jalaj S., Grimm I.S., Baron T.H. (2016). Impact of EUS-guided fine-needle biopsy sampling with a new core needle on the need for onsite cytopathologic assessment: A preliminary study. Gastrointest. Endosc..

[21] Crinò S.F., Di Mitri R., Nguyen N.Q., Tarantino I., de Nucci G., Deprez P.H., Carrara S., Kitano M., Shami V.M., Fernández-Esparrach G. (2021). Endoscopic ultrasound-guided fine-needle biopsy with or without rapid on-site evaluation for diagnosis of solid pancreatic lesions: A randomized controlled non-inferiority trial. Gastroenterology.

[22] Okusaka T., Nakamura M., Yoshida M., Kitano M., Ito Y., Mizuno N., Hanada K., Ozaka M., Morizane C., Takeyama Y. (2023). Clinical Practice Guidelines for Pancreatic Cancer 2022 from the Japan Pancreas Society: A synopsis. Int. J. Clin. Oncol..

[23] Wani S., Mullady D., Early D.S., Rastogi A., Collins B., Wang J.F., Marshall C., Sams S.B., Yen R., Rizeq M. (2015). The clinical impact of immediate on-site cytopathology evaluation during endoscopic ultrasound-guided fine needle aspiration of pancreatic masses: A prospective multicenter randomized controlled trial. Am. J. Gastroenterol..

[24] Chong C.C.N., Lakhtakia S., Nguyen N., Hara K., Chan W.K., Puri R., Almadi M.A., Ang T.L., Kwek A., Yasuda I. (2020). Endoscopic ultrasound-guided tissue acquisition with or without macroscopic on-site evaluation: Randomized controlled trial. Endoscopy.

[25] Wong T., Pattarapuntakul T., Netinatsunton N., Ovartlarnporn B., Sottisuporn J., Chamroonkul N., Sripongpun P., Jandee S., Kaewdech A., Attasaranya S. (2022). Diagnostic performance of endoscopic ultrasound-guided tissue acquisition by EUS-FNA versus EUS-FNB for solid pancreatic mass without ROSE: A retrospective study. World. J. Surg. Oncol..

[26] Kaneko J., Ishiwatari H., Sasaki K., Satoh T., Sato J., Matsubayashi H., Yabuuchi Y., Kishida Y., Yoshida M., Sayo I. (2020). Macroscopic on-site evaluation of biopsy specimens for accurate pathological diagnosis during EUS-guided fine needle biopsy using 22-G Franseen needle. Endosc Ultrasound..

[27] Kudo T., Kawakami H., Hayashi T., Yasuda I., Mukai T., Inoue H., Katanuma A., Kawakubo K., Ishiwatari H., Doi S. (2014). High and low negative pressure suction techniques in EUS-guided fine-needle tissue acquisition by using 25-gauge needles: A multicenter, prospective, randomized, controlled trial. Gastrointest Endosc..

[28] Nakai Y., Hamada T., Hakuta R., Sato T., Ishigaki K., Saito K., Saito T., Takahara N., Mizuno S., Kogure H. (2021). A Meta-analysis of Slow Pull versus Suction for Endoscopic Ultrasound-Guided Tissue Acquisition. Gut Liver..

[29] Ishikawa T., Hayakawa M., Suzuki H., Ohno E., Mizutani Y., Iida T., Fujishiro M., Kawashima H., Hotta K. (2022). Development of a Novel Evaluation Method for Endoscopic Ultrasound-Guided Fine-Needle Biopsy in Pancreatic Diseases Using Artificial Intelligence. Diagnostics.

[30] Chalhoub J.M., Hawa F., Grantham T., Lester J., Carpenter E.S., Mendoza-Ladd A., Wani S., Machicado J.D. (2024). Effect of the number of passes on diagnostic performance of EUS fine-needle biopsy of solid pancreatic masses: A systematic review and meta-analysis. Gastrointest. Endosc..

[31] Shah T., Zfass A.M. (2019). Accuracy of EUS-FNA in Solid Pancreatic Lesions: Sometimes Size Does Matter. Dig. Dis. Sci..

